# ResUbiNet: A Novel Deep Learning Architecture for Ubiquitination Site Prediction

**DOI:** 10.2174/0113892029331751240820111158

**Published:** 2024-08-27

**Authors:** Zixin Duan, Yafeng Liang, Xin Xiu, Wenjie Ma, Hu Mei

**Affiliations:** 1 Key Laboratory of Biorheological Science and Technology, Ministry of Education, College of Bioengineering, Chongqing University, Chongqing 400044, China;; 2 School of Pharmaceutical Sciences, Chongqing University, Chongqing 401331, China;; 3 College of Bioengineering, Chongqing University, Chongqing 400044, China

**Keywords:** Ubiquitination site, ResUbiNet, deep learning, prediction, ProtTrans, transformer

## Abstract

**Introduction:**

Ubiquitination, a unique post-translational modification, plays a cardinal role in diverse cellular functions such as protein degradation, signal transduction, DNA repair, and regulation of cell cycle. Thus, accurate prediction of potential ubiquitination sites is an urgent requirement for exploring the ubiquitination mechanism as well as the disease pathogenesis associated with ubiquitination processes.

**Methods:**

This study introduces a novel deep learning architecture, ResUbiNet, which utilized a protein language model (ProtTrans), amino acid properties, and BLOSUM62 matrix for sequence embedding and multiple state-of-the-art architectural components, *i.e*., transformer, multi-kernel convolution, residual connection, and squeeze-and-excitation for feature extractions.

**Results:**

The results of cross-validation and external tests showed that the ResUbiNet model achieved better prediction performances in comparison with the available hCKSAAP_UbSite, RUBI, MDCapsUbi, and MusiteDeep models.

**Conclusion:**

ResUbiNet’s integration of advanced features and architectures significantly enhances prediction performance, aiding in understanding ubiquitination mechanisms and related diseases.

## 1 INTRODUCTION

Ubiquitination represents a distinctive post-translational modification in which ubiquitin molecules are covalently attached to target proteins [1]. This intricate process plays pivotal roles in various cellular functions, including protein degradation [2, 3], signal transduction [4-6], DNA repair [7, 8], and cell cycle regulation [9, 10]. The ubiquitination cascade can be delineated into three sequential stages [11, 12]: activation, conjugation, and ligation and can be reiterated multiple times, leading to the attachment of multiple ubiquitin molecules to the target proteins. Different configurations of the ubiquitin chains can elicit different biological outcomes [13]. For instance, ubiquitination with K48-linked chains typically marks proteins for proteasomal degradation [2, 3], while K63-linked chains are involved in signal transduction [4, 6]. Recent studies have proved that abnormal ubiquitination processes are related to the occurrences of various diseases [14], such as metabolic reprogramming in cancer [15], Parkinson's disease [16], and Huntington's disease [17]. Hence, the prediction of potential ubiquitination sites is of great significance in exploring ubiquitination mechanisms and the pathogenesis of the related diseases [18].

Inspirited by traditional neural network architectures, deep learning has been widely used to unearth profound insights from biological data, assisting in unraveling the underlying biological principles [19-21], *e.g*., predicting mutations in DNA sequences [22], the functionality of DNA sequences [23, 24], miRNA precursors [25-27], and three-dimensional protein structures [28, 29]. Up to date, a few prediction models have also been developed for predicting ubiquitination sites [30]. Traditional machine learning based prediction models such as UbiPred [31], CKSAAP_UbSite [32], and hCKSAAP_UbSite [33] usually applied physicochemical properties, frequencies of k-spaced amino-acid pairs, and one-hot encoding scheme to predict ubiquitination sites. However, these amino-acid or amino-acid-pair based description methods can not characterize the position dependent relationships between ubiquitinated residues and residues flanking the ubiquitination sites.

With the development of deep learning techniques, RUBI [34] model used the probability of intrinsic disorder [35] and bi-directional recursive neural networks (BRNN) for constructing the classifier of the ubiquitination site. HubiPred [36] employed binary encoding and physicochemical properties of amino acids for establishing convolutional neural network (CNN) and recurrent neural network (RNN) prediction models of ubiquitination sites. Li *et al*. [37] proposed a MDCapsUbi model, which used one-hot encoding scheme and capsule network for constructing a multi-dimensional feature recognition approach of ubiquitination sites. Another impressive prediction model is MusiteDeep [38], which integrated multi-layer CNN and capsule network for predicting multiple post-translational modification sites including ubiquitination sites.

In spite of these pioneering researches, current prediction models of ubiquitination sites still face several limitations, including insufficient feature representation of target protein sequences, inability to capture the long-range position-dependent relationships influencing ubiquitination processes, and relatively simple network architectures. Herein, we proposed a novel deep learning model, ResUbiNet, which applied a pre-trained protein language model ProtTrans together with the physicochemical and evolutionary information for the feature representation of the amino acids upstream and downstream of the ubiquitination sites, and adopted multiple state-of-the-art architectural components like transformer, multi-kernel convolutions, and residual connection for the feature extraction of the target sequences. The results showed that the prediction performance of the ResUbiNet model was superior to that of the hCKSAAP_UbSite, RUBI, MDCapsUbi, and MusiteDeep models.

## 2 MATERIALS AND METHODS

### 2.1 Benchmark Dataset

The benchmark dataset used in this paper is derived from the research of hCKSAAP_UbSite [33], which gathered experimentally confirmed ubiquitination sites from Uniprot database as well as the available literature and proteomic assays. In the research of hCKSAAP_UbSite, a total of 9537 ubiquitinated sequences were first retrieved from 3852 proteins after removing redundant ubiquitinated sequences by Blastclust program. Then, the ubiquitinated sites (Lysine), together with 13 residues on both sides of the ubiquitination sites, were used for constructing positive samples (length = 27). Among the 9537 positive samples, 3419 from 1352 proteins were used as test samples and the remaining 6118 as training samples. For each positive sample, a negative sample (length = 27) with a non-ubiquitinated lysine in the middle was randomly selected from the same source protein. For the details of sample construction, please refer to the literature [33]. The statistics of the benchmark dataset refer to Table **1**. It is important to note that after a detailed investigation, the sample length used in this paper is 25, which is different from that of the hCKSAAP_UbSite model [33].

### 2.2 Sequence Representation

#### 2.2.1 ProtTrans

ProtTrans [39] is a natural language model aimed at understanding the language of protein sequences through self-supervised learning strategy. In this paper, the ProtT5-XL-UniRef^50^ model was used for sequence embedding, which employed Text-to-text transfer transformer [40] (T5) framework to establish a protein language model from a non-redundant UniRef^50^ database. The significant advantage of the ProtT5 model lies in its unprecedented performances, which are independent of multi-sequence alignments (MSAs) or evolutionary information. That is to say, with the ProtT5 model, we can directly extract sequence features from just a single protein sequence, bypassing the need for resource-intensive database queries. Up to date, ProtT5-XL-UniRef^50^ has been successfully applied for sequence embedding in various downstream tasks, such as the prediction of protein secondary structure and subcellular localization *etc*. Herein, a total of 1024 embedding features from ProtT5-XL-UniRef^50^ model were obtained for each sample.

#### 2.2.2 AA-index and BLOSUM62

AA-index [41] is a database aimed to collect the physicochemical and biological properties of the 20 coded amino acids. It's worthy to note that all of the amino acid properties in the AA-index database have been peer-reviewed, which ensures the data quality and dependability. Until now, there are 553 properties in AA-index database. In order to avoid redundant information, a total of 31 AA properties (Supplemental Table **S1**) from UbiPred [31] model were used in this paper. For each sample, the shape of AA property matrix is 25×31. BLOSUM62 is a widely used substitution matrix in bioinformatics [42], which has proven to be a valuable tool for characterizing protein evolution information. Herein, the dimension of BLOSUM62 matrix is 25×20 for each sample sequence. To enhance convergence during network iterations, the matrices obtained from AA-index and BLOSUM62 underwent min-max normalization. For the samples with non-standard amino acids or lengths less than 25, the missing AA properties and BLOSUM62 values were replaced by zeros.

### 2.3 Network Architecture

#### 2.3.1 The Architecture of ResUbiNet Model

**Fig. (1):**
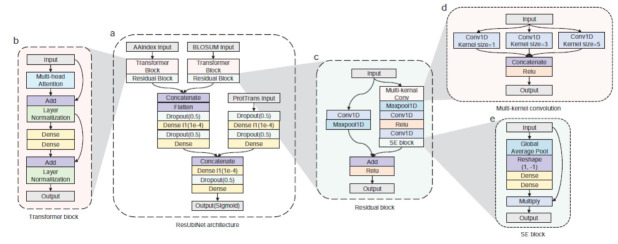
The architecture of the ResUbiNet model. (**a**) The overall architecture; (**b**): Transformer block with a multi-head attention layer and two residue connections; (**c**) Residue block with a 1-D convolutional layer and a max-pooling layer; (**d**) Multi-kernel convolution; (**e**) Squeeze-and-excitation (SE) block.

The ResUbiNet model (Fig. **1a**) has three inputs, *i.e*., AA-index, BLOSUM62, and ProtTrans features. Firstly, the AA-index and BLOSUM62 features were separately fed into a transformer block followed by a residual block. Then, the outputs of the two blocks were concatenated and processed by two dense layers with dropout treatments. After being processed by two dense layers, the ProtTrans features were concatenated with the features extracted from AAindex and BLOSUM62. Subsequently, the combined features were further processed by another two dense layers. Finally, an output layer with sigmoid function was used for predicting the probabilities of ubiquitination.

#### 2.3.2 Transformer Block

The success of the Transformer [43] model in natural language processing enlightens us to incorporate it into our prediction network [44-46]. The Transformer block (Fig. **1b**) started with a multi-head attention layer with a residue connection. The multi-head attention mechanism can capture dynamically extracted features and focus on the most important features for a given task. After data normalization, the outputs of the multi-head attention layer were then fed into a two-layer dense block with a residue connection. Finally, the normalized outputs of the dense block were fed into an output layer.

#### 2.3.3 Residual Block

The residual block (Fig. **1c**) consists of a multi-kernel convolution (Fig. **1d**), a max-pooling layer, two standard convolution layers, and a squeeze-and-excitation (SE) sub-blocks (Fig. **1e**) [47]. Rather than utilizing a fixed convolution kernel size, the multi-kernel convolution is a novel way to capture features simultaneously by using different kernel sizes. After the multi-kernel convolution, a max-pooling layer was utilized to reduce computational complexity while retaining the most significant features. Then, two standard convolutional layers were further used for extracting higher level features. Subsequently, a SE sub-block was incorporated to recalibrate feature maps by squeezing the spatial information using global average pooling. The SE sub-block allows the model to emphasize or de-emphasize certain channels based on the given task. To reconcile the matrix dimensions between the input and output of the residual block, a convolutional layer followed by a max-pooling layer was used with the kernel size of 1 and the number of filters matched with the output dimension of the residual block (Fig. **1c**). The details of the ResUbiNet network and the sub-blocks refer to Supplemental Tables (**S2-S6**).

### 2.4 Model Training

The Adam optimizer with a learning rate of 0.0005 was used for training the ResUbiNet network with a binary cross-entropy loss function and a batch size of 128. Herein, 5-fold cross-validation was performed and 5 sub-models obtained were combined to give the averaged prediction results. The ResUbiNet model was implemented by using TensorFlow package (version = 2.12.0, Python 3.11.4) and the number of trainable parameters is 399,411. The python codes and relevant data of the ResUbiNet model have been made publically available at https://github.com/Tuan-Space/ResUbiNet.

### 2.5 Model Evaluation

Accuracy (*Acc*), sensitivity (*Sn*), specificity (*Sp*), precision, *f1*-score, Matthew's correlation coefficient (*MCC*) (Eq. 1~6), the area under the receiver operating characteristic curve (*AUC*), and the area under the precision-recall curve (*AUCPR*) were used for model evaluation.


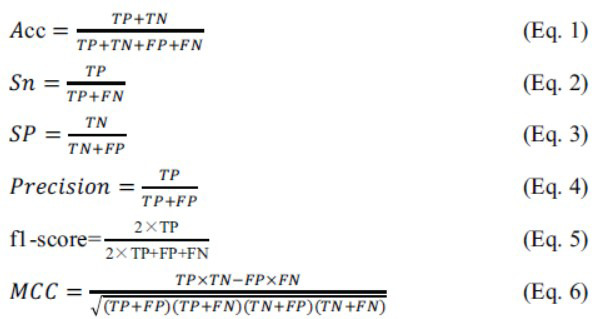


Where, *TP*, *TN*, *FP*, and *FN* denote the number of true positives, true negatives, false positives, and false negatives, respectively.

## 3 RESULTS AND DISCUSSION

### 3.1 Patten Analysis of the Ubiquitinated Sequences

**Fig. (3):**
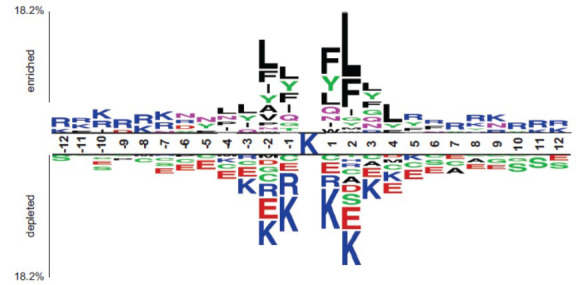
Two sample logo analysis of the amino acids adjacent to the ubiquitination sites.

Firstly, amino acid frequency analysis was performed on the positive and negative samples in the training and test sets (excluding the lysine in the middle of the sequence). From Fig. (**2**), it can be observed that F (Phe), L (Leu), R (Arg), and Y (Tyr) are more abundant in the positive samples, while C (Cys), E (Glu), K (Lys), and S (Ser) are more abundant in the non-ubiquitinated samples. Then, two sample logo [48] analysis was further performed to investigate the AA distribution of each AA position. In this study, t-test (*p* < 0.05) was used to identify the statistical significance between the positive and negative samples. For the positive samples, as shown in (Fig. **3**), L (Leu), F (Phe) and Y (Tyr) are dominant in the range of position -4 to 4. For the negative samples, K (Lys), R (Arg), C (Cys), and E (Glu) are dominant in the range of position -7 to 7. For the R (Arg), it seems dominant in a distal end of the middle lysine for the positive samples, while proximal end of the middle lysine for the negative samples. Taken together, the results indicate the preference for hydrophobic residues in the positions near to the ubiquitination sites and hydrophilic residues in the positions near to the non-ubiquitination sites. Thus, the spatial position relationships of the amino acids especially adjacent to the ubiquitination sites, play more important roles in the ubiquitination processes.

**Fig. (4):**
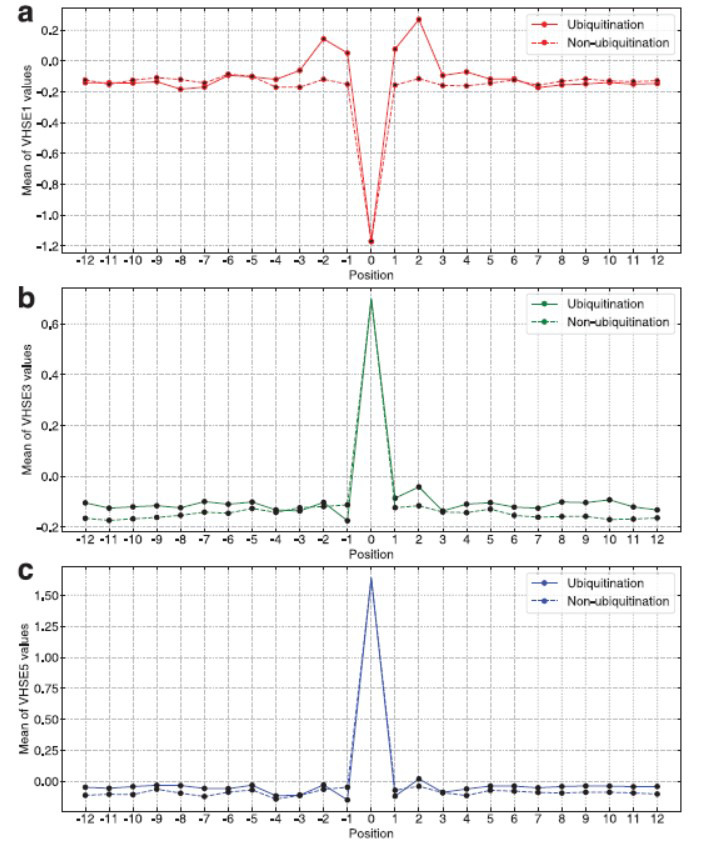
Feature distribution in each AA position of the ubiquitinated and non-ubiquitinated samples. (**a**) VHSE1 (hydrophobic property); (**b**) VHSE3(steric property); (**c**) VHSE5(electronic property).

VHSE [49] (principal component score vector of hydrophilicity, steric, and electronic properties) is derived from 50 physicochemical properties of 20 natural amino acids, of which VHSE1, VHSE3, and VHSE5 represent the first principle component scores of amino acid hydrophobic, steric, and electronic features, respectively (Supplemental Table **S7**). Herein, feature distribution of each AA position was explored by using average VHSE values of the positive and negative samples. From Fig. (**4a**), it can be observed that the AAs close to the middle lysine (Position -4 - 4) have significant hydrophobic properties (VHSE1) in the positive samples while with hydrophilic properties in the negative samples. As for the steric (VHSE3) and electronic (VHSE5) properties, it seems that bulk AAs with less negative partial charges are preferable to most of the AA positions of the positive samples in comparison with the negative samples (Fig. **4b** and **4c**).

### 3.2 The Performances of the ResUbiNet Model

To investigate the effects of sample length on prediction performances, in this paper, a total of 32 models were first established by using different sample lengths ranging from 7 to 69. In this paper, 5-fold cross-validation was employed for validating the robustness of the resulting models. The average training and validation results of 5-fold cross-validation and external testing results are shown in Fig. (**5**). It can be observed that the overall performances increased significantly with the sample lengths increasing from 7 to 25. Then, the validation and test performances tended to be stable and reached the best values at 33. With the sample lengths larger than 33, the overall performances tended to decrease. In consideration of the test performances and model complexity, in this paper, the model with a sample length of 25 was chosen as the optimal ResUbiNet model.

Figs. (**6** and **7**) show the ROC and precision-recall curves of the ResUbiNet model on the test set. It can be seen that the AUC and AUCPR values of the 5 cross-validation models are equivalent to each other. When the 5 cross-validation models are combined to give averaged prediction results, the performances on the test set are quite satisfying, of which the accuracy, sensitivity, specificity, precision, f1-score, AUC, AUCPR, and MCC values are 0.759, 0.798, 0.720, 0.740, 0.768, 0.833, 0.809, and 0.520, respectively.

### 3.3 Comparison with Other Machine Learning Methods

For comparison purpose, SVM [50], Random Forest (RF) [51], K-Nearest Neighbors (KNN) [52], XGBoost [53], Deep Neural Network (DNN) [54], Convolutional Neural Network (CNN) [55], and Long Short-Term Memory (LSTM) [56] were also used to establish prediction models by using the same training and test datasets. Fig. (**8**) shows the ROC curves of the resulting models on the test set. It's obvious that the ResUbiNet model outperforms the other 7 machine learning models. The details and prediction performances of the 7 models are shown in Supplemental Table **S8** and **S9**.

### 3.4 Comparison with the Available Prediction Models

We further compared the prediction performances of the ResUbiNet model with that of the hCKSAAP_UbSite [33], RUBI [34], MDCapsUbi [37], MusiteDeep [38], CNN_Binary [57], CNN_Property [57], and multi-layer CNN [58] models. Herein, the performances of the hCKSAAP_UbSite and RUBI models on the test set were obtained from the literature [33, 34] and that of the MDCapsUbi, MusiteDeep, CNN_Binary, CNN_Property, and multi-layer CNN models were derived from our retraining results by using the same dataset. The prediction performances of the models above are shown in Table **2**. It can be observed that the performances of the ResUbiNet model are significantly better than that of the hCKSAAP_UbSite, MDCapsUbi, RUBI, CNN_Binary, CNN_Property, MusiteDeep, and multi-layer CNN models, although the sensitivity value is lower than that of the MusiteDeep model. Besides, it is worthy to note that the ResUbiNet model shows more balanced prediction performances on the positive and negative samples in comparison with the MDCapsUbi, CNN_Binary, CNN_Property and MusiteDeep models. In addition, the sample length used in the ResUbiNet model is only 25, which is the smallest length among the 8 models.

Herein, the ubiquitination samples from dbPTM database [59] were further used for evaluating the prediction performances of the ResUbiNet model. Since the length of the ubiquitination samples in the dbPTM dataset is 21, we first reconstructed the ubiquitination samples with 25 residues according to the source protein sequences from UniProt [60] and ubiquitination site annotations. After removing non-human ubiquitination sequences, the samples without centered lysine (K) and duplicates with the hCKSAAP_UbSite dataset, 2023 ubiquitinated samples and 2023 randomly selected non-ubiquitinated samples were obtained. The prediction accuracy and AUC values on this independent test set are 0.709 and 0.774, which indicate good generalization capability of the ResUbiNet model.

## CONCLUSION

As a crucial post-translational modification, ubiquitination is closely related to diverse biological functions and processes. Therefore, an accurate method for predicting ubiquitination sites is of great value for understanding the ubiquitination mechanism as well as the related disease pathogenesis. In this paper, a novel deep learning architecture, ResUbiNet, was proposed for predicting ubiquitination sites based on 31 AA properties, BLOSUM62, and embedding features derived from ProtTrans model. The results showed that the ResUbiNet model achieved better prediction performances in comparison with the hCKSAAP_UbSite, RUBI, MDCapsUbi, MusiteDeep, CNN_ Binary, CNN_Property, and multi-layer CNN models. From the results, it can be indicated that the integration of the embedding features of the ProtTrans language model with AA physicochemical and evolutionary information is an efficient way to promote prediction capability. Also, the transformer, multi-kernel convolution, residual connection, and squeeze-and-excitation architectures benefit the feature extraction of both local and global contextual information. Although the ResUbiNet model has been successful, there is still room for further improvement. For example, in the sample construction process, the influences of the lengths of residues upstream and downstream of the ubiquitination sites on the model prediction performance should be considered respectively. Also, the integration of the ubiquitination databases encompassing a broader range of species, such as *Mus musculus*, *Saccharomyces cerevisiae*, and *Arabidopsis thaliana* could further enhance the model's generalizability. Such efforts will not only validate the model's performances across diverse biological contexts but also contribute to a deeper understanding of the ubiquitination mechanisms across different species.

Looking ahead, the ever-evolving bioinformatics and deep learning methods present both challenges and opportunities for the prediction of ubiquitination sites. The broader goal is to understand the functional implications of the ubiquitination modifications, their interactions with other cellular processes, and their role in disease onset and progression. The integration of ubiquitination site prediction with other related fields and interdisciplinary collaboration would bring significant achievements to the prediction of ubiquitination site.

## Figures and Tables

**Fig. (2):**
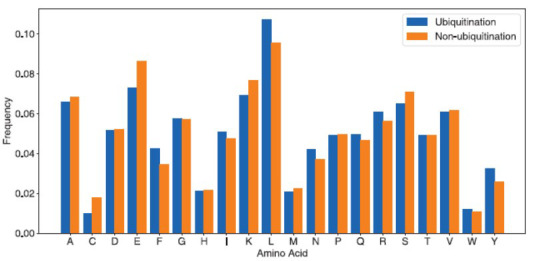
The occurrence frequencies of the amino acids adjacent to the ubiquitination sites.

**Fig. (5):**
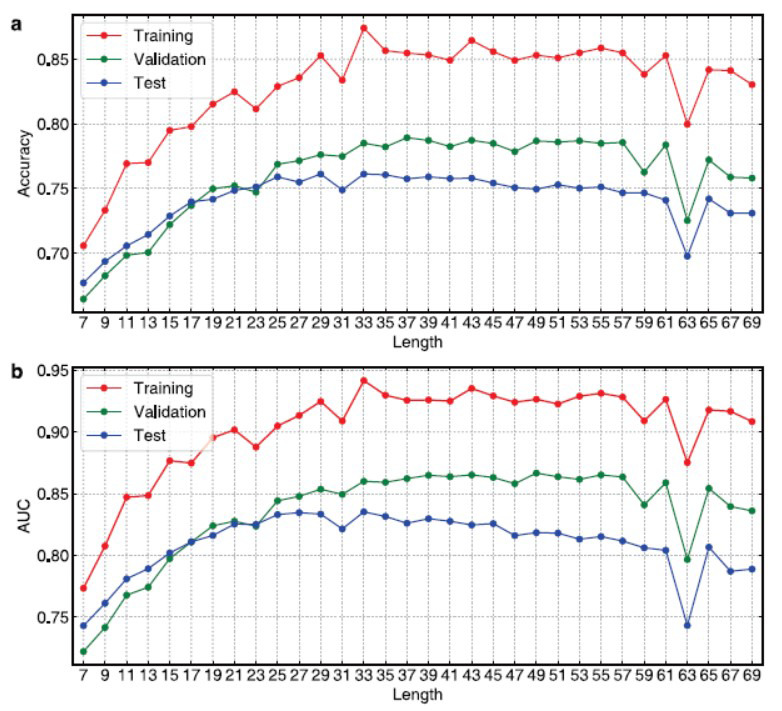
The model performances established by using different sample lengths. (**a**) Accuracy; (**b**) AUC. The performances on the training and validation sets were the average results of 5-fold cross-validation. The test performances were the average prediction results of the 5 cross-validation models.

**Fig. (6):**
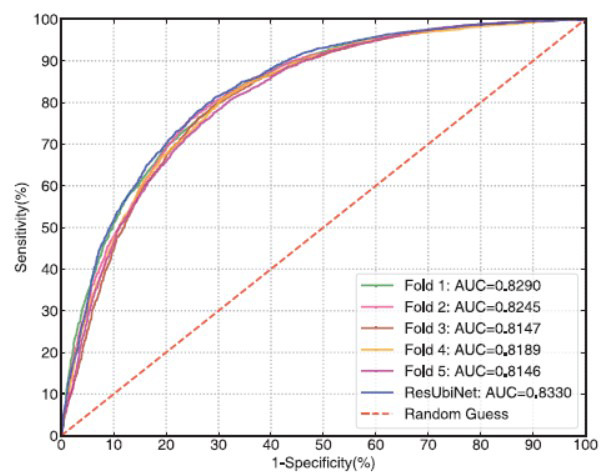
The ROC curves on the test set.

**Fig. (7):**
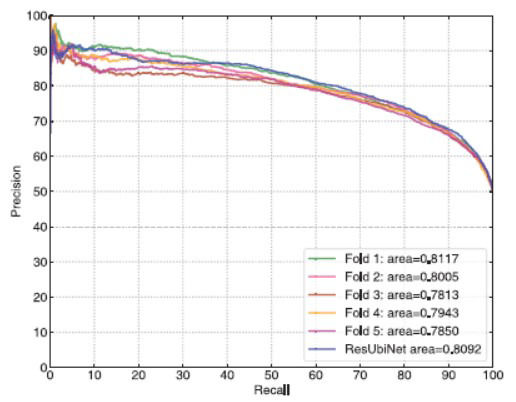
The precision-recall curves on the test set.

**Fig. (8):**
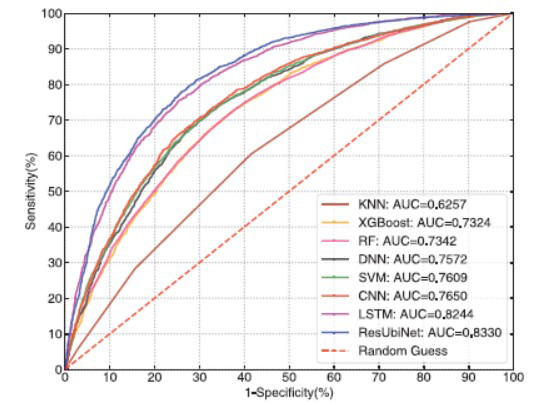
The ROC curves of different classifiers on the test set.

**Table 1:** The benchmark dataset used for establishing the ResUbiNet model.

**Dataset**	**Positive**	**Negative**	**Total**
Training/validation set	6118	6118	12236
Test set	3419	3419	6838
Total	9537	9537	19074

**Table 2:** The prediction performances of the ResUbiNet model in comparison with the other 7 models.

**Model**	**Acc**	**Sn**	**Sp**	**Precision**	**F1**	**AUC**	**MCC**	**Length**
hCKSAAP_UbSite	NA	NA	NA	NA	NA	0.757 [33]	NA	27
MDCapsUbi	0.605	0.833	0.377	0.572	0.678	0.654	0.236	69
RUBI	NA	NA	NA	NA	NA	0.820 [34]	NA	27
CNN_Binary	0.698	0.953	0.442	0.631	0.759	0.834	0.460	41
CNN_Property	0.701	0.951	0.450	0.634	0.761	0.827	0.464	41
MusiteDeep	0.730	0.876	0.583	0.678	0.764	0.825	0.480	33
Multi-layer CNN	0.736	0.755	0.716	0.724	0.740	0.808	0.472	31
ResUbiNet	0.759	0.798	0.720	0.740	0.768	0.833	0.520	25

## Data Availability

The authors confirm that the data supporting the findings of this research are available within the article.

## LIST OF ABBREVIATIONS

BRNN: Bi-directional Recursive Neural Networks
CNN: Convolutional Neural Network
DNN: Deep Neural Network
KNN: K-Nearest Neighbors
LSTM: Long Short-Term Memory
MSAs: Multi-sequence Alignments
RF: Random Forest
RNN: Recurrent Neural Network
SE: Squeeze-and-Excitation
