# Supplementary material for: ResUbiNet: A Novel Deep Learning Architecture for Ubiquitination Site Prediction
Source: Curr Genomics. 2024 Aug 27;26(4):302–11. doi: 10.2174/0113892029331751240820111158 (PMC12606654; doi:10.2174/0113892029331751240820111158)
Supplement: Supplementary file 1 [file CG-26-4-302_SD1.pdf]

Supplementary Material

ResUbiNet: A Novel Deep Learning Architecture for Ubiquitination Site Prediction

Zixin Duan[1,2], Yafeng Liang[2], Xin Xiu[3], Wenjie Ma[3] and Hu Mei[1,3,*]

[1]Key Laboratory of Biorheological Science and Technology, Ministry of Education, College of Bioengineering, Chongqing University, Chongqing 400044, China; [2]School of Pharmaceutical Sciences, Chongqing University, Chongqing 401331, China; [3]College of Bioengineering, Chongqing University, Chongqing 400044, China

Table S1. The 31 amino acid properties selected from the AAindex database.

AAindex Identity	Description
NADH010102	Hydropathy scale based on self-information values in the two-state model of 9% accessibility
BROC820102	Retention coefficient in HFBA
MEIH800102	Average reduced distance for side chain
LEVM780101	Normalized frequency of alpha-helix, with weights
GUYH850104	Apparent partition energies calculated from Janin index
CORJ870101	NNEIG index
RACS770102	Average reduced distance for side chain
GEOR030108	Linker propensity from helical (annotated by DSSP) dataset
HARY940101	Mean volumes of residues buried in protein interiors
GRAR740102	Polarity
GUYH850105	Apparent partition energies calculated from Chothia index
MEIH800103	Average side chain orientation angle
KRIW790102	Fraction of site occupied by water
LEVM780106	Normalized frequency of reverse turn, unweighted
BULH740102	Apparent partial specific volume
FAUJ880101	Graph shape index
PUNT030102	Knowledge-based membrane-propensity scale from 3D_Helix in MPtopo databases
HUTJ700103	Entropy of formation
EISD840101	Consensus normalized hydrophobicity scale
CEDJ970105	Composition of amino acids in nuclear proteins (percent)
ZIMJ680102	Bulkiness
CEDJ970103	Composition of amino acids in membrane proteins (percent)
CHOC760103	Proportion of residues 95% buried
CEDJ970102	Composition of amino acids in anchored proteins (percent)
ROSM880102	Side chain hydropathy, corrected for solvation
BROC820101	Retention coefficient in TFA
FAUJ830101	Hydrophobic parameter pi

NAKH920101	AA composition of CYT of single-spanning proteins
ZHOH040102	The relative stability scale extracted from mutation experiments
NAKH900101	AA composition of total proteins
QIAN880129	Weights for coil at the window position of -4

Table S2. The architecture and parameters of Transformer block.

Layer	Layer Type	Parameters	Connection
1	Multi-head Attention	Number of Heads: 2, Key Dimension: 32	Input Layer
2	Layer Normalization	Epsilon: 1e-6	Input Tensor Add the Output of Layer 1
3	Dense	Units: 32, Activation: ReLU	Output of Layer 2
4	Dense	Units: Same as Input Layer Dimension	Output of Layer 3
5	Layer Normalization	Epsilon: 1e-6	The Output of Layer 2 Add the Output of Layer 4

Table S3. The architecture and parameters of Multi-kernel convolution.

Layer	Layer Type	Parameters	Connection
1	1D Convolution	Filters: 32, Kernel Size: 1, Padding: Same	Input Layer
2	1D Convolution	Filters: 32, Kernel Size: 3, Padding: Same	Input Layer
3	1D Convolution	Filters: 32, Kernel Size: 5, Padding: Same	Input Layer
4	Concatenation	-	Outputs of Layers 1-3
5	Activation	Activation: Relu	Output of Layer 4

Table S4. The architecture and parameters of SE block.

Layer	Layer Type	Parameters	Connection
1	Global Average Pooling 1D	-	Input Tensor
2	Reshape Layer	Target Shape: (1, filters)	Output of Layer 1
3	Dense	Units: filters // 8, Activation: Relu, Kernel Initializer: He Normal Initialization, Use Bias: False	Output of Layer 2
4	Dense	Units: filters, Activation: Sigmoid, Kernel Initializer: He Normal Initialization, Use Bias: False	Output of Layer 3
5	Multiply Layer	-	Input Tensor and Output of Layer 4

The parameter ‘filters’ represents the last dimension of the input tensor of SE block.

Table S5. The architecture and parameters of Residual block.

Number	Layer Type	Parameters	Connection
1	Multi-Kernel Convolutional Block	Filters: 32	Input Layer
2	Max Pooling 1D	-	Output of Layer 1
3	1D Convolutional Layer	Filters: 32, Kernel Size: 3, Padding: Same	Output of Layer 2
4	Activation Layer	Activation: Relu	Output of Layer 3
5	1D Convolutional Layer	Filters: 32, Kernel Size: 3, Padding: Same	Output of Layer 4
6	SE Block	-	Output of Layer 5

7	1D Convolutional Layer	Filters: 32, Kernel Size: 1, Padding: Same	Input Layer
8	Max Pooling 1D	-	Output of Layer 7
9	Add Layer	-	Outputs of Layers 6 & 8
10	Activation Layer	Activation: Relu	Output of Layer 9

Table S6. The architecture and parameters of ResUbiNet.

Pathway	Number	Layer Type	Parameters	Connection
AAindex	1	Input Layer	Shape: (25,31)	-
AAindex	2	Transformer Block	-	Output of Layer 1
AAindex	3	Residual Block	-	Output of Layer 2
BLOSUM	1	Input Layer	Shape: (25,20)	-
BLOSUM	2	Transformer Block	-	Output of Layer 1
BLOSUM	3	Residual Block	-	Output of Layer 2
AA&BLO	1	Concatenation	-	Outputs of AAindex and BLOSUM Pathways
AA&BLO	2	Flatten Layer	-	Output of Layer 1
AA&BLO	3	Dropout	Rate: 0.5	Output of Layer 2
AA&BLO	4	Dense	Units: 256, Activation: Relu, Regularizer: L1(1e-4)	Output of Layer 3
AA&BLO	5	Dropout	Rate: 0.5	Output of Layer 4
AA&BLO	6	Dense	Units: 16, Activation: Relu	Output of Layer 5
ProtTrans	1	Input Layer	Shape: (1024,)	-
ProtTrans	2	Dropout	Rate: 0.5	Output of Layer 1
ProtTrans	3	Dense Layer	Units: 128, Activation: Relu, Regularizer: L1(1e-4)	Output of Layer 2
ProtTrans	4	Dropout	Rate: 0.5	Output of Layer 3
ProtTrans	5	Dense	Units: 16, Activation: Relu	Output of Layer 4
Final	1	Concatenation	-	Outputs of AA&BLO Pathway and Prot Pathway
Final	2	Dense	Units: 128, Activation: Relu, Regularizer: L1(1e-4)	Output of Layer 1
Final	3	Dropout	Rate: 0.5	Output of Layer 2
Final	4	Dense	Units: 16, Activation: Relu	Output of Layer 3
Final	5	Output Layer	Units: 1, Activation: 'sigmoid'	Output of Layer 4

Table S7. The VHSE1, VHSE3, and VHSE5 values of 20 coded amino acids.

Index	A	R	N	D	C	Q	E	G	H	I
VHSE1	0.15	-1.47	-0.99	-1.15	0.18	-0.96	-1.18	-0.2	-0.43	1.27
VHSE3	-1.35	1.24	-0.37	-0.41	-0.46	0.18	0.1	-2.63	0.37	0.3
VHSE5	0.02	1.55	-0.55	-2.68	0	0.09	-2.16	-0.53	0.51	0.3
Index	L	K	M	F	P	S	T	W	Y	V
VHSE1	1.36	-1.17	1.01	1.52	0.22	-0.67	-0.34	1.5	0.61	0.76

VHSE3	0.26	0.7	0.43	0.96	-0.5	-1.07	-0.55	1.79	1.17	-0.17
VHSE5	0.22	1.64	0.23	0.25	-0.01	-0.32	-0.06	0.75	0.53	0.22

Table S8. The details of the 7 machine learning models.

Model: "SVM"
C=1.0, kernel='rbf'

Model: " RF "
n_estimators=100, criterion='gini', max_depth=None, min_samples_split=2, min_samples_leaf=1, min_weight_fraction_leaf=0.0

Model: "KNN "
n_neighbors=5, weights='uniform', algorithm='auto' , leaf_size=30, p=2

Model: " XGBoost"
objective='binary:logistic', base_score=0.5, booster='gbtree'

Model: "DNN"

```
____________________________________________________________________________________
 Layer (type)                Output Shape        Param #     Connected to
====================================================================================
 input_1 (InputLayer)        [(None, 25, 31)]     0           []
 input_2 (InputLayer)        [(None, 25, 20)]     0           []
 concatenate (Concatenate)   (None, 25, 51)       0           ['input_1[0][0]',  'input_2[0][0]']
 flatten (Flatten)           (None, 1275)         0           ['concatenate[0][0]']
 input_3 (InputLayer)        [(None, 1024)]       0           []
 concatenate_1 (Concatenate) (None, 2299)         0           ['flatten[0][0]',  'input_3[0][0]']
 dense (Dense)               (None, 128)          294400      ['concatenate_1[0][0]']
 dense_1 (Dense)             (None, 1)            129         ['dense[0][0]']
====================================================================================
Total params: 294,529
Trainable params: 294,529
Non-trainable params: 0
____________________________________________________________________________________
```

Model: "CNN"

```
____________________________________________________________________________________
 Layer (type)                Output Shape        Param #     Connected to
====================================================================================
 input_4 (InputLayer)        [(None, 25, 31)]     0           []
 input_5 (InputLayer)        [(None, 25, 20)]     0           []
 concatenate_2 (Concatenate) (None, 25, 51)       0           ['input_4[0][0]',  'input_5[0][0]']
 conv1d (Conv1D)             (None, 25, 32)       4928        ['concatenate_2[0][0]']
 flatten_1 (Flatten)         (None, 800)          0           ['conv1d[0][0]']
 input_6 (InputLayer)        [(None, 1024)]       0           []
 concatenate_3 (Concatenate) (None, 1824)         0           ['flatten_1[0][0]',  'input_6[0][0]']
 dense_2 (Dense)             (None, 1)            1825        ['concatenate_3[0][0]']
====================================================================================
Total params: 6,753
Trainable params: 6,753
Non-trainable params: 0
____________________________________________________________________________________
```

Model: "LSTM"

```
____________________________________________________________________________________
 Layer (type)                Output Shape        Param #     Connected to
====================================================================================
 input_7 (InputLayer)        [(None, 25, 31)]     0           []
 input_8 (InputLayer)        [(None, 25, 20)]     0           []
 concatenate_4 (Concatenate) (None, 25, 51)       0           ['input_7[0][0]', 'input_8[0][0]']
```

```
lstm (LSTM)                (None, 25, 32)      10752        ['concatenate_4[0][0]']
flatten_2 (Flatten)              (None, 800)         0            ['lstm[0][0]']
input_9 (InputLayer)          [(None, 1024)]        0             []
concatenate_5 (Concatenate)     (None, 1824)         0        ['flatten_2[0][0]',  'input_9[0][0]']
dense_3 (Dense)                (None, 1)          1825        ['concatenate_5[0][0]']
==================================================================================
Total params: 12,577
Trainable params: 12,577
Non-trainable params: 0
__________________________________________________________________________________
```

Table S9. The prediction performances of the 7 machine learning models on the test set.

Method	ACC	Sn	Sp	Precision	F1	AUC	MCC
SVM	0.6984	0.7230	0.6739	0.6892	0.7057	0.7609	0.3974
RF	0.6755	0.6970	0.6540	0.6683	0.6823	0.7342	0.3513
KNN	0.5948	0.6043	0.5853	0.5930	0.5986	0.6257	0.1896
XGBoost	0.6752	0.7180	0.6323	0.6614	0.6885	0.7324	0.3517
DNN	0.6983	0.7277	0.6689	0.6873	0.7069	0.7572	0.3973
CNN	0.7008	0.7382	0.6634	0.6868	0.7116	0.7650	0.4027
LSTM	0.7464	0.7818	0.7110	0.7301	0.7551	0.8244	0.4941
